# Trait anxiety mediates between emotion dysregulation and core psychopathology in borderline personality disorder

**DOI:** 10.1192/j.eurpsy.2022.945

**Published:** 2022-09-01

**Authors:** E. Kot, P. Grzegorzewski, B. Kostecka, J. Radoszewska, K. Kucharska

**Affiliations:** 1 Institute of Psychiatry and Neurology, Department Of Neuroses, Personality Disorders, And Eating Disorders, Warsaw, Poland; 2 Medical University of Warsaw, Ii Department Of Psychiatry, Warsaw, Poland; 3 University of Warsaw, Faculty Of Psychology, Warsaw, Poland; 4 Cardinal Stefan Wyszynski University in Warsaw, Institute Of Psychology, Warsaw, Poland

**Keywords:** borderline personality disorder, Emotion dysregulation, trait anxiety, depressieve symptoms

## Abstract

**Introduction:**

Findings from previous studies have implicated various forms of emotion dysregulation (EDys), including difficulties in emotion regulation, as important for the development and maintenance of borderline personality disorder (BPD). In addition, comorbid anxiety and depressive psychopathology has been found to contribute to the severity of BPD symptoms in this disorder.

**Objectives:**

This study aimed at extending extant research on the above issues by testing the mediational role of comorbid psychopathology (i.e., trait anxiety and depressive symptoms) in the relationship between EDys (specifically, difficulties in emotion regulation) and BPD symptoms in BPD patients.

**Methods:**

64 BPD female inpatients completed the Emotion Dysregulation Scale, STAI, CESD–R, and BPD Checklist.

**Results:**

BPD symptoms were statistically significantly and strongly positively associated to EDys (*r_s_* = .55, *p* < .001) and to trait anxiety (*r_s_* = .52, *p* < .001). EDys and trait anxiety predicted the severity of BPD symptoms, *R^2^* = 0.41, *F*(2, 61) = 20.90, *p* < .001. The examination of the indirect effect revealed a significant mediation, in which the association between EDys and BPD symptoms was mediated by trait-anxiety, *B* = 0.37, SE = 0.18, 95% CI = [0.10, 0.78]. However, the direct effect of EDys on BPD symptoms remained significant, *B* = 0.81, SE = 0.24, 95% CI = [0.32, 1.30].

**Conclusions:**

The severity of BPD symptoms is associated with EDys and with trait anxiety. Moreover, our findings show that the latter partially mediates the link between EDys and BPD symptoms, which suggests that trait anxiety may contribute to the severity of BPD symptoms.

**Disclosure:**

No significant relationships.

